# Translational Research in Ovarian Cancer

**DOI:** 10.3390/cancers12123676

**Published:** 2020-12-08

**Authors:** Maria Flavia Di Renzo, Giorgio Valabrega

**Affiliations:** 1 Department of Oncology, University of Torino, 10124 Torino, Italy; 2 Candiolo Cancer Institute, FPO-IRCCS, 10060 Candiolo TO, Italy

Ovarian cancer is still the most lethal gynecologic malignancy with a median five-year survival of 48%, including the less malignant and early diagnosed cases [1]. Indeed, evidence from molecular biology and genetics shows that epithelial ovarian cancer includes not only the most frequent and lethal high-grade serous ovarian cancer, characterized by p53 mutation and, frequently, by homologous recombination deficiency, but also rare histotypes, such as mucinous, clear cells, low-grade, and borderline cancers. The latter show defined pathologic and molecular features (e.g., HER2 overexpression and dysregulation of the PI3K–AKT pathway) and peculiar clinical behavior (i.e., chemoresistance). Unfortunately, most ovarian cancers are diagnosed at an advanced stage and only marginal improvements in overall survival are achieved by standard treatments [2]. 

Therefore, in translational studies, important issues are drug resistance, targeted therapies, and immunotherapy. In the context of drug resistance, ovarian cancer clonal evolution and tumor heterogeneity may be crucial and, as a direct consequence, liquid biopsies may become essential to monitor cancer progression [3]. The introduction of inhibitors targeting PARP in current clinical practice as first line treatment for naïve and relapsed cases has considerably improved the outcome of patients with homologous recombination defective ovarian cancer [4,5]. However, there is still an alarming gap in the fundamental knowledge and understanding of primary and acquired resistance to PARP inhibitors. The complexity of ovarian cancer, in comparison with other solid cancers, also translates into slower development of other targeted therapies: only antiangiogenic drugs are established tools in the treatment of ovarian cancer with modest outcomes [6]. 

Another important issue is the role of immunotherapy in ovarian cancer. Although a strong preclinical and translational rationale supports in-depth investigation, preliminary data from trials applying immune checkpoint inhibitors in different clinical settings have shown marginal or absent activity. Much work is therefore needed to identify molecular, pathologic, and clinical features that may predict sensitivity to checkpoint inhibitors and to bring other immunotherapeutic approaches to the clinic, such as cell-based therapies [7].

In conclusion, this Special Issue welcomes articles that illustrate and stimulate the rapid advances that are taking place in the area of ovarian cancer based on translational research. We are interested in studies on the molecular mechanisms of development and of relapse of ovarian cancer (including rare histotypes), mode of action and resistance to targeted drugs, new targets for personalized therapy of the disease, and models to investigate novel therapeutic approaches transferable to clinical trials.
